# Towards Universal Structure-Based Prediction of Class II MHC Epitopes for Diverse Allotypes

**DOI:** 10.1371/journal.pone.0014383

**Published:** 2010-12-20

**Authors:** Andrew J. Bordner

**Affiliations:** Department of Molecular Pharmacology and Experimental Therapeutics, Mayo Clinic, Scottsdale, Arizona, United States of America; John Innes Centre, United Kingdom

## Abstract

The binding of peptide fragments of antigens to class II MHC proteins is a crucial step in initiating a helper T cell immune response. The discovery of these peptide epitopes is important for understanding the normal immune response and its misregulation in autoimmunity and allergies and also for vaccine design. In spite of their biomedical importance, the high diversity of class II MHC proteins combined with the large number of possible peptide sequences make comprehensive experimental determination of epitopes for all MHC allotypes infeasible. Computational methods can address this need by predicting epitopes for a particular MHC allotype. We present a structure-based method for predicting class II epitopes that combines molecular mechanics docking of a fully flexible peptide into the MHC binding cleft followed by binding affinity prediction using a machine learning classifier trained on interaction energy components calculated from the docking solution. Although the primary advantage of structure-based prediction methods over the commonly employed sequence-based methods is their applicability to essentially any MHC allotype, this has not yet been convincingly demonstrated. In order to test the transferability of the prediction method to different MHC proteins, we trained the scoring method on binding data for DRB1*0101 and used it to make predictions for multiple MHC allotypes with distinct peptide binding specificities including representatives from the other human class II MHC loci, HLA-DP and HLA-DQ, as well as for two murine allotypes. The results showed that the prediction method was able to achieve significant discrimination between epitope and non-epitope peptides for all MHC allotypes examined, based on AUC values in the range 0.632–0.821. We also discuss how accounting for peptide binding in multiple registers to class II MHC largely explains the systematically worse performance of prediction methods for class II MHC compared with those for class I MHC based on quantitative prediction performance estimates for peptide binding to class II MHC in a fixed register.

## Introduction

The binding of peptide fragments of extracellular proteins to class II MHC is a critical step in activating a helper T cell-mediated immune response. The discovery of such peptide epitopes has several important biomedical applications. For example, peptide epitopes from pathogen antigens that bind to multiple MHC allotypes present in a population are needed for developing vaccines with broad protective immunity. Also, class II MHC has a role in autoimmune diseases, as specific class II MHC alleles have been found to be either positively or negatively associated with many autoimmune diseases including type 1 diabetes [1], [2], [3], rheumatoid arthritis [4], multiple sclerosis [5], [6], celiac disease [7], and narcolepsy [8], [9]. Peptide binding specificities for risk-associated alleles could help identify new causative autoantigens or help investigate mechanistic hypotheses such as competitive capture by alternative binding registers [10], [11]. They can also help find possible mechanisms for the protective effects of other alleles. In addition, such information can guide the search for therapeutic inhibitors that block autoantigen binding by the responsible MHC allotype. Finally, class II epitopes show promise as an immunotherapy for the treatment of allergies [12], [13], [14], [15], [16], [17] so that information on these epitopes could potentially be used to design effective allergy therapies.

In spite of these promising potential applications, experimental information on peptide-MHC binding specificities is limited in coverage since class II MHC is highly polymorphic and the space of peptide sequences is enormous. Computational methods can assist by predicting peptide-MHC binding affinities that can later be experimentally validated. Such prediction methods broadly fall into two categories, sequence-based and structure-based, each with complementary advantages and disadvantages. Sequence-based methods are fast but require a large quantity of experimental binding data for the MHC type of interest. Although slower, structure-based methods are more general and can potentially be applied to any class II MHC type, including experimentally uncharacterized ones.

Sequence-based methods use patterns in peptide sequences with known binding affinities to a particular MHC allotype in order to predict binding affinities. Such methods have been developed for peptide binding to class II MHC using a wide variety of fitting techniques including partial least squares (PLS) [18], [19], Gibbs sampling [20], linear programming [21], Support Vector Machines (SVMs) [22], [23], [24], kernel methods [25], non-linear optimization with a regularization penalty [26], or a combination of data fitting techniques [27]. A few methods can even make predictions for closely related MHC types not used for training [28], [29], [30], basically by interpolating between prediction models for the few experimentally characterized MHC types based on limited structural information about shared MHC residues or pockets. However, no sequence-based method can make predictions for an MHC allotype that is significantly different from experimentally characterized MHC allotypes so that it does not share most of its specificity-determining residues. A survey of the available experimental peptide-MHC binding data from the Immune Epitope Database (IEDB) [31] shows that only a minute fraction of the hundreds to thousands of MHC allotypes for each locus have sufficient experimental data to train a sequence-based model. For example, considering human MHC, only about 14 HLA-DR allotypes, 5 HLA-DP allotypes, and 5 HLA-DQ allotypes currently have sufficient experimental data. These data permit the construction of multi-type sequence-based prediction models that cover of the majority of known HLA-DR allotypes but only a small fraction of HLA-DP and HLA-DQ allotypes [30]. Structure-based prediction methods, such as the one described here, can address this shortcoming and potentially make predictions for any MHC allotype based on the universal physical principles of intermolecular interactions. Such methods can be used to suggest novel peptide epitopes for under-characterized or uncharacterized MHC allotypes for subsequent experimental testing.

In contrast with sequence-based methods, comparatively little work has been done to explore structure-based methods for predicting peptide binding affinities to class II MHC. Davies *et al.* 2003 [32] built homology models of DRB1*0301, DRB1*0401, and DRB1*1101 using a DRB1*0101 template structure and docked peptides into these MHC models using simulated annealing optimization with the AMBER force field in explicit water. The resulting peptide-MHC interaction energy of the complex was then used to discriminate binders from non-binders. The study reported prediction accuracies comparable with the contemporaneous SYFPEITHI and TEPITOPE sequence-based methods, as evaluated for small test sets of 22–30 peptides. Another study by Schafroth and Floudas [33] docked individual amino acids into five binding pockets in DRB1*0101 and compared the predicted qualitative binding preferences of each pocket with experimental results. In a more recent study by Tong *et al.* [34], the authors docked peptides into a DQA1*0301/DQB1*0302 MHC structure from the Protein Data Bank (PDB) using a four-step procedure. The docking procedure consisted of rigid body docking of terminal core fragments, central loop closure, constrained all-atom refinement of the core segment, and extension of the core segment. Peptide binding affinities were predicted using a linear combination of the hydrophobic, entropic, and electrostatic components of the interaction free energy with optimized weights. Finally, during the course of this work, a new study by Zhang et al. [35] appeared, which describes a comparison of three different structure-based prediction methods using a common data set of peptides binding to DRB1*0101. The three methods were (1) complex structure prediction using MODELLER and scoring using a statistical residue pair potential, (2) molecular dynamics simulation in explicit water of all possible single residue mutants of a single peptide epitope binding the MHC followed by derivation of a position specific scoring matrix (PSSM) based on the average interaction energy with Poisson-Boltzmann surface area (PBSA) implicit solvation over 100 MD snapshots, and (3) a PSSM derived from the number of intermolecular residue contacts in available X-ray structures of peptide-DRB1*0101 complexes. The three methods yielded comparable AUC values of 0.682, 0.667, and 0.621, respectively. While the first two methods are potentially applicable to other MHC allotypes, the latter method requires a sufficient number of experimental peptide-MHC structures for the allotype of interest in order to derive the PSSM and so is likely limited to DRB1*0101 at present. One novel aspect of our study, not addressed by these previous studies, is a demonstration of the generality of the prediction method by applying it to a wide variety of MHC allotypes with distinct peptide binding specificities. This is arguably the single most important advantage of structure-based methods over sequence-based ones and so is crucial to test.

The binding of peptides to class I and class II MHCs differ in several respects. Class I MHC binds short peptide fragments (∼8–11 residues), generally derived from intracellular proteins, whereas class II MHC binds longer fragments (∼15–25 residues) of extracellular proteins. The reason for this difference is evident from available X-ray structures of peptide-MHC complexes. The class I MHC peptide binding cleft is closed at both ends and binds the peptide partly through conserved hydrogen bonds to the peptide backbone at the N- and C-termini. Thus the peptide backbone assumes a conserved conformation at the termini and bulges out from the cleft in the center. In contrast, the class II MHC peptide-binding cleft is open at both ends so that the peptide binds in an extended polyproline II conformation so that both termini can extend beyond the cleft. The core 9-mer peptide segment contacting the MHC assumes a common backbone conformation due to conserved hydrogen bonds to backbone atoms along its entire length. For both MHC classes, the conserved hydrogen bond interactions of the MHC with the peptide backbone contribute to high affinity binding for a large number of different peptides while interactions with the peptide side chains determine the characteristic binding specificity of each MHC allotype.

In accordance with these differences, the prediction strategies for class II MHC differ from those for class I MHC. Most importantly, all possible binding registers of the peptide, defined by the core 9-mer segment contacting the MHC, must be considered in a class II MHC prediction method. Furthermore, the experimental data only provides the overall peptide binding affinity and not information on the predominant binding register. Our method accounts for this ambiguity by docking all possible 9-mer segments and predicting the peptide as a binder if any segment is predicted to bind strongly, and otherwise as a non-binder. A machine learning classifier is used to identify whether or not each 9-mer segment is a binder based on interaction energy components calculated from the docking solution. Because experimental data on the binding registers is unavailable, the classifier is trained on a balanced set of 9-mer binders and non-binders predicted using a sequence-based prediction method [26]. Also, in order to reduce costly conformational sampling and obtain a native-like peptide backbone conformation, the peptide backbone is restrained to be near the native conformation during the docking simulation.

We first tested and optimized the peptide-MHC docking procedure by comprehensive self-docking and cross-docking followed by comparison of the results with all available human and murine peptide-MHC complex structures in the PDB. Next we trained the machine learning classifier on binding data for DRB1*0101 and made predictions for peptide binding to multiple dissimilar MHC allotypes in order to test the transferability of the prediction method. We also assessed the accuracy of the prediction method on the separate task of predicting predominant peptide binding registers and compared the results with known binding registers inferred from available X-ray structures of peptide-MHC complexes. Based on a few simple assumptions, we derived rough estimates of the prediction performance for peptides binding class II MHC in a particular fixed register and found that the calculated values were similar to that previously obtained for peptide binding to class I MHC, in which only one binding mode is possible. This provides an explanation for the previously noted overall lower accuracy of prediction methods for peptide binding to class II MHC compared to those for class I MHC. Finally, we discuss the implications of the results for general prediction of peptide-MHC binding affinities and possible future improvements.

## Methods

### Overview of the structure-based prediction procedure

The goal of the prediction procedure is to determine whether a particular peptide is a binder (IC_50_ <500 nM) or a non-binder (IC_50_ ≥500 nM) for the MHC allotype of interest. This is accomplished by three steps: (1) docking of all possible 9-mer core segments from the peptide into the MHC protein to predict the structures of the bound complexes, (2) machine-learning based scoring to predict the 9-mer binding affinities based on the structures from step (1), and (3) calculating the final binding score as the maximum score over all 9-mer segments.

### Peptide-MHC docking

**Figure 1:**
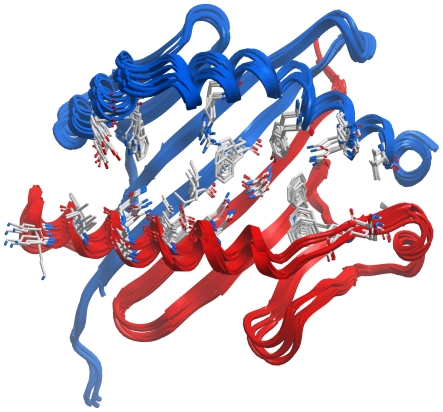
Similar HLA-DRB1*0101 peptide-contacting residue conformations observed in X-ray structures. All HLA-DRB1*0101 peptide-MHC complexes were superimposed by aligning the backbones of the MHC peptide binding domains (chain A residues 1–82 and chain B residues 1–92). MHC chains A and B are shown in red and blue ribbon representation, respectively. The MHC residues contacting the bound peptides, shown in stick representation, generally adopt similar conformations despite contacting different peptides. Much of the small side chain deviations are due to imperfect alignment of the MHC backbone atoms. This observation motivates the use of a rigid potential map representation of the MHC binding cleft in the docking procedure for efficiency rather than computationally expensive sampling of the contacting portion of the MHC.

The docking method is similar to that previously employed for class I MHC [36], with the main difference being the use of modified peptide backbone constraints. First, an all-atom flexible model of the peptide with neutral N- and C-terminal groups was docked into a grid potential representation of the MHC peptide-binding cleft. The MHC structure from the highest resolution peptide-MHC structure in the PDB was used (see **Tables 1** and **2**). Grid potentials have the advantage of being dramatically more computationally efficient than all-atom sampling of MHC side chains while allowing implicit flexibility through smoothed van der Waals interactions that allow limited steric clashes. Furthermore, an alignment of peptide-MHC complex structures for the MHC allotype with the most structures, HLA-DRB1*0101, shown in **Figure 1**, demonstrates that the MHC side chains contacting the peptides undergo little conformational change upon binding different peptides. While some conformational differences in the MHC peptide-binding cleft must occur in order to accommodate the binding of different peptides, this limited experimental evidence suggests that these differences are relatively small.

**Table 1:** Peptide – human class II MHC docking results.

Peptide-MHC complex structure	Docked peptide sequence	Backbone RMSD (Å)	All-atom RMSD (Å)	Core backbone RMSD (Å)	Core all-atom RMSD (Å)	Contacting peptide residue numbers
DRB1*0101 (1KLU)						
1KLU*	EL**IGTLNAAKV**PAD	0.26	1.21	0.23	0.75	−1–1,3,4,6–10
1AQD	SD**WRFLRGYHQ**Y	0.86	2.34	0.86	2.58	0–5,7–10
2G9H	PK**YVKQNTLKL**A	0.92	1.53	0.98	1.62	−1–7,9,10
1KLG	EL**IGILNAAKV**P	0.29	1.67	0.30	0.90	−1–1,3,4,6–10
1SJE	PEVIPMFSALSE	0.75	2.13	0.61	1.84	−1–10
1T5W	AA**YSDQATPLL**L	0.88	1.21	0.94	1.28	−1–4,6–10
2FSE	AG**FKGEQGPKG**E	0.86	2.17	0.81	1.91	1,2,4,7,10
DRB1*0301 (1A6A)						
1A6A*	SK**MRMATPLLM**Q	0.26	0.97	0.23	0.76	−1–10
DRB1*0401 (1J8H)						
1J8H*	PK**YVKQNTLKL**A	0.39	0.56	0.28	0.51	−1–10
DRB1*1501 (1BX2)						
1BX2*	PV**VHFFKNIVT**P	0.24	0.78	0.21	0.83	−1–6,9,10
DRB3*0101 (2Q6W)						
2Q6W*	A**WRSDEALPL**G	0.20	0.62	0.21	0.63	0–9
DRB3*0301 (3C5J)						
3C5J*	QV**IILNHPGQI**S	0.32	1.56	0.22	1.16	−1,1–6,8–10
DRB5*0101 (1FV1)						
1FV1*	HF**FKNIVTPRT**P	0.34	1.55	0.24	0.94	0–4,6
1H15	GV**YHFVKKHVH**E	0.32	0.49	0.29	0.50	0–4,6
DQA1*0501/DQB1*0201 (1S9V)						
1S9V*	LQ**PFPQPELPY**	0.27	0.88	0.22	0.30	−1–4,6,7,9
DQA1*0102/DQB1*0602 (1UVQ)						
1UVQ*	MN**LPSTKVSWA**A	0.26	0.44	0.26	0.26	−1–4,6–10
DQA1*0301/DQB1*0302 (2NNA)						
2NNA*	SG**EGSFQPSQE**N	0.30	1.15	0.22	0.64	−1,1,3–10
1JK8	LV**EALYLVCGE**R	0.90	1.73	0.56	0.74	0–7,9,10
DPA1*0103/DPB1*0201 (3LQZ)						
3LQZ*	RK**FHYLPFLPS**T	0.15	1.04	0.12	1.13	0–4,6,7,9,10
**Self-docking median RMSDs**		0.26	0.97	0.22	0.75	
**Cross-docking median RMSDs**		0.86	1.70	0.71	1.45	

Self-docking results are indicated by asterisks. The PDB ID for the MHC structure used in docking is shown in parentheses for each allotype. All-atom RMSDs were calculated for all non-hydrogen atoms in the peptide residues that contact the MHC in the experimental structure. The core RMSDs include only peptide residues P1–P9 (underlined in the peptide sequence) whereas the other values include all simulated residues from P-1 to P10. The highest resolution MHC structure, in parentheses, was used for each allotype. The RMSD values were calculated after aligning the MHC structures. Consecutive residue numbers in the last column are denoted by a numeric range.

**Table 2:** Peptide – murine class II MHC docking results.

Peptide-MHC complex structure	Docked peptide sequence	Backbone RMSD (Å)	All-atom RMSD (Å)	Core backbone RMSD (Å)	Core all-atom RMSD (Å)	Contacting peptide residue numbers
**H2-IA^b^ (1MUJ)**						
1MUJ*	PVSK**MRMATPLLM**QA	0.25	0.88	0.26	0.58	−1–10
1LNU	FE**AQKAKANKA**VD	0.70	1.83	0.74	1.96	−1–10
**H2-IA^d^ (2IAD)**						
2IAD*	*see footnote*					
1IAO	I**SQAVHAAHA**E	0.48	1.58	0.42	1.03	1–10
**H2-IA^g7^ (1ES0)**						
1ES0*	YE**IAPVFVLLE**Y	0.20	2.44	0.21	0.29	−1–10
1F3J	MK**RHGLDNYRG**Y	0.83	1.79	0.75	1.27	−1–2,4–8,10
3CUP	KK**MREIIGWPG**G	0.88	2.24	0.87	1.62	−1–5,7,8
**H2-IA^k^ (1IAK)**						
1IAK*	ST**DYGILQINS**R	0.33	1.18	0.33	1.03	−1–2,4–10
**H2-IA^u^ (1K2D)**						
1K2D*	SR**GGASQYRPS**Q	0.17	0.73	0.17	0.60	0,3–10
**H2-IE^k^ (1FNG)**						
1FNG*	KV**ITAFNEGLK**	0.44	1.34	0.33	1.24	−1–6,8,9
1FNE	KV**ITAFNDGLK**	0.51	1.29	0.45	1.20	0–4,6–9
1IEB	RM**VNHFIAEFK**	0.83	1.67	0.83	1.70	0–4,6–9
1R5V	DL**IAYPKAATK**F	0.91	2.27	0.77	0.99	−1–7,9,10
1KT2	DL**IAYLKQATK**	0.72	1.38	0.65	1.17	−1–4,6–9
1R5W	DL**IAYFKAATK**F	1.43	2.42	0.93	1.20	−1–7,9,10
1KTD	DL**IAYLKQASA**K	1.50	3.06	0.61	1.14	−1–10
**Self-docking median RMSDs**		0.25	1.18	0.26	0.60	
**Cross-docking median RMSDs**		0.83	1.81	0.75	1.20	

See the **Table 1** caption for details. The correct peptide sequence for PDB entry 2IAD was unknown since it differs between the reference and the PDB file so docking of this peptide was not performed.

Docking was performed using biased-probability Monte Carlo global optimization [37] of a physical energy function using the ICM program (Molsoft LLC). The energy function is a sum of the intramolecular all-atom energy of the peptide calculated using the ECEPP/3 force field [38], [39], [40], the interaction energy of the peptide and the MHC calculated using grid potentials, and a harmonic restraint potential on the peptide backbone. Five types of grid potentials were used for the non-hydrogen atom van der Waals (E_Cvw_), hydrogen atom van der Waals (E_Hvw_), hydrogen bond (E_hb_), electrostatics (E_el_), and hydrophobic (E_hp_) components of the peptide-MHC interaction energy. These potentials were precomputed on a rectilinear grid with 0.5 Å spacing containing the peptide and peptide-binding domain of the MHC. Potential values at arbitrary points were calculated using linear interpolation of values at the nearest grid points. E_Cvw_ and E_Hvw_ were calculated as the smoothed van der Waals (vdW) interaction energy, with a cutoff value 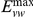 = 3 kcal/mol at zero separation, between corresponding probe atoms at grid points and the MHC protein [41]. The smooth vdW potential further reduces the extreme sensitivity of the vdW energy to small conformational changes. The hydrogen bond and hydrophobic energies were calculated as described in Ref. [41] and the electrostatic energy was calculated using a distance-dependent dielectric ε = 4r. Weights multiplying the grid potentials were optimized to yield the lowest average RMSD from among the 5 lowest energy docking solutions accumulated during each Monte Carlo run. All possible combinations of weight values between 0.0 and 5.0 in 0.5 increments were tried. As expected, the optimal weights were similar to those we previously obtained for peptide-class I MHC docking using a different optimization protocol [36]. The intramolecular peptide energy (E_peptide_) was calculated with the ECEPP/3 force field and included a truncated vdW potential with 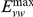 = 7 kcal/mol, the distance-dependent dielectric electrostatic term, hydrogen bond, torsional potentials, and a side chain entropic term proportional to the fractional SASA [37]. Finally, a harmonic restraint potential,
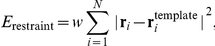
between corresponding peptide backbone atoms in the modeled peptide and those in the template peptide-MHC complex structure was used to limit the conformational sampling space, and so speed up convergence, while insuring that the final docking solution has a backbone conformation similar the conserved conformation observed in X-ray structures. All peptide backbone atoms between P-1 to P9 were included in the restraint potential based on the extent of the conserved backbone structure observed in X-ray structures. The restraint weight value w = 1.0 kcal/(mol Å^2^) was found to yield the best cross-docking results and so used in all subsequent docking simulations. The final energy function used for docking was then
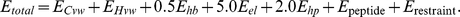
The ICM Monte Carlo simulations were run for a total 5×10^7^ function calls using a temperature parameter of 700K. This required an average simulation time of approximately 10 hours on a 3 GHz Opteron processor.

### All-atom structure optimization and interaction energy evaluation

The lowest energy docking solutions were then subjected to all-atom structure optimization using a more accurate physical energy function and the interaction energy components of the final structure used as input to the machine learning scoring method. First the MHC protein was replaced by an all-atom model and the docking solution structure refined by local optimization of an all-atom energy function that included ECEPP/3 energy terms (vdW smoothed with 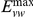 = 7 kcal/mol, hydrogen bond, and torsion potentials), generalized Born solvation electrostatics with ECEPP/3 atomic charges and ε_in_ = 4, a non-polar solvation term proportional to the SASA with constant 0.012 kcal/(mol Å^2^), a side chain entropy term (described above), and the same peptide backbone restraint potential used for grid potential docking. The goal of this procedure was not to improve the docked structure, as local optimization led to only minor variations in the initial grid potential docking solution, but rather to reduce steric clashes and so yield accurate values for the interaction energy components using a more detailed all-atom model of the MHC protein and a more realistic implicit solvation model.

### Machine learning based scoring and binding affinity prediction

The peptide binding affinities were predicted by first predicting whether or not each 9-mer fragment binds; if at least one was predicted to bind then the peptide was classified as a binder, otherwise it was classified as a non-binder. A Random Forest binary classifier [42] trained on interaction energy components and 20 residue type counts was used to predict whether or not each 9-mer fragment bound to the MHC allotype of interest. The interaction energy components included van der Waals, hydrogen bond, electrostatics, non-polar solvation, and entropy contributions calculated from the final refined docking solution as the difference in these energy terms between the peptide-MHC complex and the isolated peptide and MHC in their bound conformations. In principle, the optimized conformation of the isolated peptide could be used, however this did not improve the binding affinity predictions (data not shown). Two empirical residue potentials, the Betancourt-Thirumalai contact potential [43] and DFIRE-SCM side chain centroid potential [44], were also included in the input data. Both potentials were included since their correlation was quite low (Pearson correlation coefficient = 0.45 for DRB1*0101 data) so that they provided largely independent information. The DFIRE-SCM potential was refit using the latest non-redundant structures from the culled PDB data set [45] with 30% sequence identity, 2.0 Å resolution, and 0.25 R-factor cutoffs. The inclusion of residue type counts can be physically justified on the basis of a random coil model of the free energy for the isolated peptide, in which each residue makes an additive contribution based on its type. Finally, we note that, unlike sequence-based models, no properties that depend on the residue positions (P1–P9) are used. This would be expected to ruin the transferability of the prediction model to multiple highly dissimilar MHC types, which is the primary motivation for the structure-based model.

As mentioned above, one challenge of peptide binding affinity predictions for class II MHC is that only the binding affinities and not the binding registers are generally known experimentally. This was solved by training the Random Forest classifier on sequence-based predictions for individual 9-mer fragments. Binary predictions (binder/non-binder) were made using our RTA method [26] for all 9-mers in the set of 1725 DRB1*0101 peptide sequences using the same 500 nM IC_50_ cutoff as used for complete peptides. In order to construct a balanced training data set, input data for all 9-mer fragment binders and an equal number of randomly selected non-binders were included. A Random Forest with 2000 trees and 5 variables/tree was used since it yielded the best prediction performance for DRB1*0101, as assessed on out-of-bag training set data. Because the two Random Forest parameters were selected using training set data alone, prediction results for the test sets are expected to accurately estimate the prediction performance for novel peptides and MHC allotypes. Because Random Forest performance converges with an increasing number of trees [42], the minimum number of trees required for a reasonably converged result was chosen for computational speed. Also, as previous observed [42], we found that the prediction accuracy did not change much as the remaining model parameter, the number of variables per tree, was varied.

### Experimental peptide binding affinity data

Experimental peptide-MHC binding affinity data were used for training the scoring method and evaluating the prediction performance. Binding data for DRB1*0101, DQA1*0501/DQB1*0201, H2-IA^b^, and H2-IA^d^ were downloaded from the Immune Epitope Database (IEDB) [31]. All quantitative data obtained by either radioactivity or fluorescence competition binding assays were included. The DPA1*0103/DPB1*0201 binding data were obtained from Ref. [46] and included known epitopes as well as a set of overlapping peptides spanning *Phleum pratense* antigens. Single residue mutation data, employed in the SAAS analysis, was excluded due to its limited sequence diversity. Finally, data for similar peptide sequences with >40% sequence identity were removed using CD-HIT [47] in order to obtain non-redundant data sets. Also any sequences in the DRB1*0101 training set with 100% identity to any sequence included in the other test sets were removed. Even though the binding affinities of a peptide to two such different MHC allotypes are expected to be uncorrelated, this was done to reduce any possible systematic bias in amino acid composition between the training and test sets. The total numbers of binding data in each set, corresponding to a particular MHC allotype, are given in **Table 3**.

**Table 3:** Prediction performance for different MHC types using a Random Forest classifier trained on DRB1*0101 peptide binding data.

			AUC		
MHC type	Number of peptides	Number of unique 9-mer segments	Peptide	Estimated Core 1	Estimated Core 1–2
DRB1*0101	1725	12858	0.707	0.876	0.854
DQA1*0501/DQB1*0201	236	1783	0.683	0.909	0.900
DPA1*0103/DPB1*0201	219	1602	0.821	0.939	0.925
H2-IA^b^	361	2572	0.671	0.875	0.858
H2-IA^d^	106	898	0.632	0.707	0.681

The estimated core AUC values assume either exactly one strongly binding 9-mer segment per binding peptide (“Estimated Core 1”) or either 1 or 2 strongly binding 9-mer segments with a 70% and 30% probability of occurrence for each strongly binding peptide (“Estimated Core 1–2”).

### Estimating the prediction performance for individual MHC-binding segments

The usual prediction performance for complete peptides binding to class II MHC is assessed by directly comparing the predictions with experimental binding affinity data. We assessed prediction performance by calculating the area under the Receiver Operating Characteristic curve (AUC) for predictions on separate test set data. It is also informative to carry out a similar performance analysis for the binding prediction of individual 9-mer segments of the complete peptides since it is known that only such short segments actually contact the MHC and so contribute to the overall binding affinity. Because the experimental data only provides binding affinities of complete peptides and not information on which segments bind the MHC, the prediction performance for segments must be estimated based on a particular prediction model. Our model predicts that a peptide is a binder if and only if at least one 9-mer segment is predicted as a binder by the Random Forest classifier. In order to find the relation between the prediction performance for the 9-mer segments and the complete peptides an additional assumption about the number of binding 9-mer segments in each binding peptide is required. We examine two possibilities: (1) exactly one binding segment is always present or (2) either one or two binding segments are present in each binding peptide. Previous analyses using sequence-based prediction models by us [26] and others [34] suggest that multiple segments often contribute to the overall binding affinity however case (1) is also a reasonable first approximation. Next, we derive the AUC for segment predictions in these two cases. It should be emphasized that because of the necessary approximations, the segment AUC values should only be considered semi-quantitative estimates. However, as will be seen below, the observation that they are uniformly higher than the AUC values for complete peptides is robust and results from combining multiple 9-mer segment predictions to arrive at a binding prediction for each peptide.

### AUC estimate for predicting 9-mer segments assuming exactly one binding segment

The AUC is the area under the Receiver Operating Characteristic (ROC) curve. This curve plots the true positive rate, tpr, versus the false positive rate, fpr, as the score cutoff is varied. Our goal then is to express tpr_seg_ and fpr_seg_ for individual 9-mer segments in terms of tpr_pep_ and fpr_pep_ for complete peptides at a common Random Forest score cutoff value. The AUC for 9-mer segments can then be calculated from these values as the area under the curve.

We first define the conditional probabilities that a peptide or fragment is predicted to be a binder (P_pred_) or non-binder (N_pred_), given that it actually is a binder (P_exp_) or non-binder (N_exp_). The fpr is then an estimate of p(P_pred_|N_exp_) and the tpr is an estimate of p(P_pred_|P_exp_). Given that even a single predicted binding fragment in a non-binding peptide results in an incorrect prediction that the peptide is a binder we have
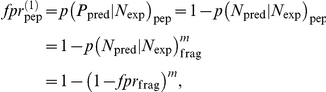
in which *m* is the number of 9-mer fragments per peptide. The average peptide length in the data sets is 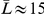 so that 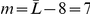. Likewise the peptide true positive rate is
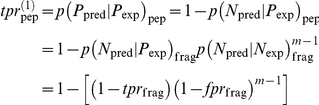
Finally, solving for the fragment fpr and tpr gives
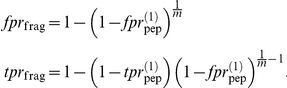
These equations are then used to obtain fpr_frag_ and tpr_frag_ at each cutoff value and so calculate the AUC for predicting individual 9-mer fragments.

### AUC estimate for predicting 9-mer segments assuming up to two binding segments

A similar argument gives the tpr if *exactly two* binding segments are present in binding peptides
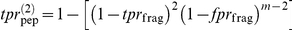
so that the total peptide tpr for either one or two binding segments in a binder is
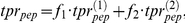
in which f_1_ and f_2_ are the fractions of binding peptides with exactly one or two binding segments, respectively. We obtained a rough estimate of these fractions by counting the number of binding 9-mer fragments per binding peptide using the sequence-based RTA model applied to HLA-DRB1*0101 data. This gave the values 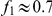 and 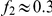, which were used in subsequent calculations. The fpr is the same as in the previous case, *i.e.* 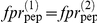. The quadratic relation between {fpr_pep_, tpr_pep_} and {fpr_frag_, tpr_frag_} may then be solved to obtain expressions for the latter quantities in term of the former ones. As in the previous case, these relations are then used to calculate the AUC for predicting individual 9-mer fragments.

## Results

### Peptide-MHC docking accuracy

**Figure 2:**
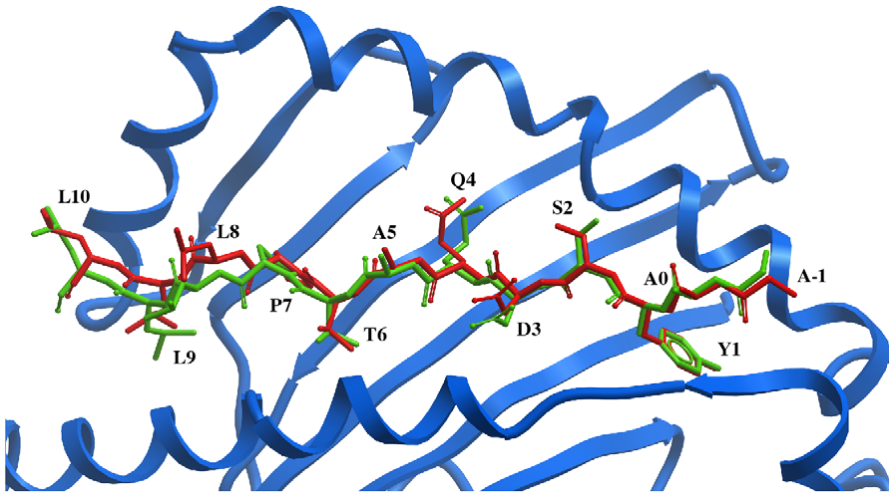
Example of a successful peptide-MHC cross-docking solution. This structure was obtained by docking the peptide from PDB entry 1T5W (AAYSDQATPLLL) into a grid potential model of the HLA-DRB1*0101 MHC structure from PDB entry 1KLU, which has a different peptide bound. The RMSD of the peptide backbone is 0.88 Å and the RMSD of the contacting residues is only 1.21 Å compared with the X-ray structure. The peptide docking solution is shown in red and the experimental peptide structure is shown in green.

The accuracy of the peptide-MHC docking procedure was determined by docking peptides from all peptide-MHC complex structures in the PDB into the representative bound MHC structure from the highest resolution complex starting with the peptide in a fully extended conformation. In other words, no *a priori* information on the peptide conformation beyond the backbone restraints described above was used for docking. The resulting RMSDs of the backbone and all contacting residue atoms are shown in **Table 1** for human MHCs and **Table 2** for murine MHCs. The cross-docking results, in which a peptide is docked into an MHC structure with a different peptide bound, best reflect the expected accuracy for docking novel peptides. Self-docking, or redocking, is considerably easier since the MHC residues in the peptide-binding cleft are already in the exact bound conformation and so it does not account for any structural rearrangements of the MHC peptide-binding cleft. In addition to prediction results for the full-length peptide, results are also given for the 9-mer core segment since they are most relevant to the binding affinity prediction method in which all 9-mer segments are docked. **Figure 2** shows an example of a successful cross-docking solution in which the RMSD of peptide residues contacting the MHC is only 1.21 Å despite the fact that the original MHC structure had a different peptide bound.

It is apparent from **Tables 1** and **2** that almost all peptide side chains contact the MHC protein (non-hydrogen atom separation ≤4 Å) and so determine binding specificity to varying degrees. In fact, each peptide residue between P-1 and P10 contacts the MHC in multiple structures. This implies that prediction methods which only account for peptide residues that bind into canonical MHC pockets, *e.g.* peptide residues P1, P4, P6, and P9 for HLA-DRB1*0101, may be missing some peptide-MHC interactions that contribute to binding. The method described here only includes core residues P1–P9. This was done because variations in the peptide backbone of flanking residues makes their prediction more difficult, as reflected in the generally higher RMSDs for the longer segments from P-1 to P10 compared with the 9-mer core segments shown in **Tables 1** and **2**. Because most peptides have side chain contacts to the MHC outside of the 9-mer core, a promising area of future study is to attempt to improve the docking accuracy for full length peptides and so possibly improve the binding affinity prediction accuracy.

We examined in detail the solution from docking the 1AQD peptide into the 1KLU MHC structure since it stands out with significantly higher RMSD than any of the other docking solutions. The high RMSD was predominantly due to two misplaced arginine residues at P2 (RMSD = 4.1 Å) and P5 (RMSD = 3.5 Å) whose side chains have only minimal interactions with the MHC in the native structure, with one hydrogen bond each between the guanidinium group and the MHC backbone, making it difficult to predict their conformations correctly. Likewise, the errors in the other docking solutions with high core RMSDs, namely the 1LNU-1MUJ, 2FSE-1KLU, and 1SJE-1KLU peptide-MHC complexes, are mainly due to misplaced large residues at non-pocket positions with minimal MHC interactions in the native structure: lysine at P8 (3.4 Å), lysine at P2 (3.3 Å), and phenylalanine at P5 (3.9 Å), respectively. Such residues at positions outside of the usual pockets are able to make energetically favorable contacts because of their large size. Presumably such peptide residues with few intermolecular interactions contribute relative little to the overall peptide-MHC binding affinity so that such docking errors do not dramatically reduce binding affinity prediction performance.

### Peptide-MHC binding affinity predictions

**Figure 3:**
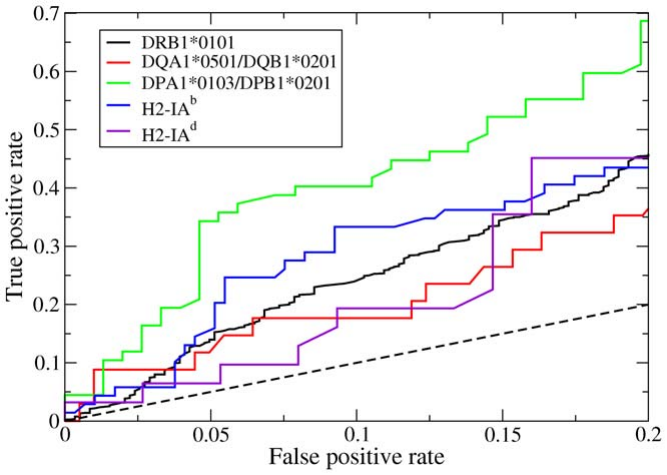
ROC curves for peptide binding affinity predictions using the data sets described in **Table 3**. Only the region with low false positive rate, which is most relevant in practice, is shown.

For all allotypes except DRB1*0101, predictions were made for all peptides in the respective data sets using the Random Forest classifier trained on DRB1*0101 binding data. Because it was used for training, the prediction performance for DRB1*0101 was assessed by 10-fold cross-validation. The prediction performance results, as measured by the AUC values, for three human allotypes (DRB1*0101, DQA1*0501/DQB1*0201, and DPA1*0103/DPB1*0201) and two murine allotypes (H2-IA^b^ and H2-IA^d^) are shown in **Table 3**. The corresponding ROC curves for the relevant low false positive rate range, 0≤ fpr ≤0.2, is shown in **Figure 3**. The particular allotypes used for testing were chosen for two reasons: (1) adequate peptide binding data was available for evaluating the prediction results and (2) they have very different peptide binding specificities so that the results reflect the generality of the prediction method for multiple MHC allotypes. As expected, the prediction performance for DRB1*0101 was quite high since data for this allotype were used for training. Although those particular results do not measure the method's generality, the results are encouraging since the cross-validation procedure insured separate training and test data sets. Interestingly, an even higher accuracy was achieved for DPA1*0103/DPB1*0201, demonstrating the excellent transferability to a distinct HLA-DP locus. The high accuracy for this allotype may be related to our observations that both the RTA (data not shown) and MultiRTA [30] sequence-based prediction methods yielded generally higher accuracy for HLA-DP allotypes compared with HLA-DR allotypes. However, we find no obvious explanation for the higher HLA-DP prediction accuracy.

### Relative contributions of input variables

**Figure 4:**
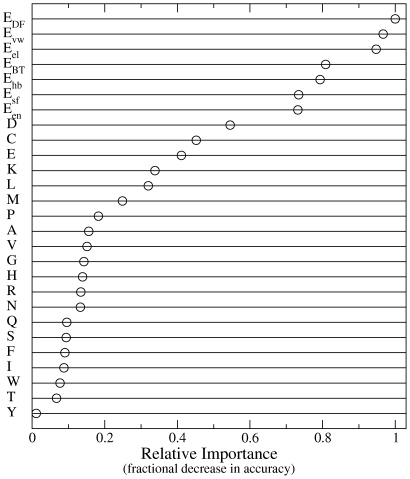
Relative importance of the Random Forest input properties to the overall binding affinity prediction accuracy. These results show that the interaction energy terms contribute the most to the prediction performance.

Although the Random Forest classifier is not readily interpretable as is, for example, a linear model, the relative importance of each input variable to the overall prediction accuracy can be estimated. This is accomplished by calculating the reduction in the prediction accuracy for so-called out-of-bag data, not included in the bootstrap training sample, upon permuting the values for the variable of interest [42]. The results of this analysis using the balanced DRB1*0101 data set are shown in **Figure 4**. This shows that the empirical and physical interaction energy components make larger contributions than any of the residue type counts. This is reasonable on physical grounds since the free energies of the isolated peptides are partially accounted for by the interaction energy components in addition to the residue type counts. The analysis also shows that the van der Waals interactions, which favor close packing between the peptide and MHC protein, is the most important energy component. The high importance of the empirical potentials, which are dominated by hydrophobic van der Waals interactions, also supports this interpretation.

We also fit a type of generalized linear model, a logistic regression classifier, to the DRB1*0101 training data using only the five physical energy components. This was done to obtain an easily interpretable prediction model in order to examine it in terms of physical interactions. The optimal weights are shown in **Table 4**. One important result is that all weights have the same correct sign so that favorable interactions for each energy component contribute to an overall favorable peptide-MHC binding affinity. Furthermore, all weights were shown to be statistically significant (at a 5% level), *i.e.* are expected to be non-zero and so contribute to the overall prediction.

**Table 4:** Logistic regression coefficients for a model trained only on DRB1*0101 binding energy components.

Variable	Coefficient value (standard error)	Significance
ΔE_vw_	−0.112 (0.00838)	<2.0×10^−16^
ΔE_hb_	−0.108 (0.0143)	3.7×10^−14^
ΔE_el_	−0.0203 (0.0102)	4.7×10^−2^
ΔE_en_	−0.643 (0.0482)	<2.0×10^−16^
ΔE_sf_	−0.339 (0.0546)	5.0×10^−10^
Intercept	−10.1 (0.844)	<2.0×10^−16^

All coefficients are statistically significant (p<5%) and are negative, as expected from physical considerations.

### Peptide binding register predictions

We also compared the predicted predominant peptide binding register, for which the core peptide had the strongest binding affinity, with the one observed in available X-ray structures of peptide-MHC complexes. This provides an independent test of the prediction method. The results for individual complexes are shown in **Table 5**. Overall, binding registers were correctly predicted for 14/19 (74%) of the human complexes and 12/15 (80%) of the murine complexes. If restricted only to the cross-docking results then 6/9 (67%) of the human complexes and 7/10 (70%) of the murine complexes were correctly predicted.

**Table 5:** Peptide binding register predictions compared with all human and mouse peptide-MHC X-ray structures.

MHC allotype	MHC PDB entry	Peptide PDB entry	Peptide sequence	Rank of core segment/Number of core segments
DRB1*0101	1KLU	1KLU*	GEL**IGTLNAAKV**PAD	1/7
DRB1*0101	1KLU	1AQD	VGSD**WRFLRGYHQ**YA	2/7
DRB1*0101	1KLU	1DLH, 2G9H	PK**YVKQNTLKL**AT	1/5
DRB1*0101	1KLU	1KLG	GEL**IGILNAAKV**PAD	1/7
DRB1*0101	1KLU	1SJE	PE**VIPMFSALS**EGATP	1/8
DRB1*0101	1KLU	1T5W	AA**YSDQATPLL**LSPR	1/7
DRB1*0101	1KLU	2FSE	AG**FKGEQGPKG**EPG	1/6
DRB1*0301	1A6A	1A6A*	KPKPPKPCSK**MRMATPLLM**QALPM	1/16
DRB1*0401	1J8H	1J8H*	PK**YVKQNTLKL**AT	1/5
DRB1*0401	1J8H	2SEB	QY**MRADQAAGG**LR	2/5
DRB1*1501	1BX2	1BX2*	ENPV**VHFFKNIVT**PR	1/7
DRB3*0101	2Q6W	2Q6W*	A**WRSDEALPL**GS	1/4
DRB5*0101	1FV1	1FV1*	NPVVHF**FKNIVTPRT**PPPSQ	3/12
DRB5*0101	1FV1	1H15	GGV**YHFVKKHVH**ES	1/6
DQA1*0501/DQB1*0201	1S9V	1S9V*	QLQ**PFPQPELPY**	1/4
DQA1*0102/DQB1*0602	1UVQ	1UVQ*	MN**LPSTKVSWA**AV	1/5
DQA1*0301/DQB1*0302	2NNA	2NNA*	QQYPSG**EGSFQPSQE**NPQ	2/10
DQA1*0301/DQB1*0302	2NNA	1JK8	SHLV**EALYLVCGE**RG	5/7
DPA1*0103/DPB1*0201	3LQZ	3LQZ*	RK**FHYLPFLPS**T	1/4
H2-IA^b^	1MUJ	1MUJ*	PVSK**MRMATPLLM**QA	1/7
H2-IA^b^	1MUJ	1LNU	FE**AQKAKANKA**VD	1/5
H2-IA^d^	2IAD	1IAO	I**SQAVHAAHA**EI	1/4
H2-IA^g7^	1ES0	1ES0*	YE**IAPVFVLLE**YVT	1/6
H2-IA^g7^	1ES0	1F3J	AMK**RHGLDNYRG**YSL	1/7
H2-IA^g7^	1ES0	3CUP	KK**MREIIGWPG**GSGG	1/7
H2-IA^k^	1IAK	1IAK*	TDGST**DYGILQINS**RW	1/8
H2-IA^u^	1K2D	1K2D*	SR**GGASQYRPS**QR	1/5
H2-IE^k^	1FNG	1FNG*	GKKV**ITAFNEGLK**	1/5
H2-IE^k^	1FNG	1FNE	GKKV**ITAFNDGLK**	1/5
H2-IE^k^	1FNG	1IEB	RDRM**VNHFIAEFK**	1/5
H2-IE^k^	1FNG	1KT2	RDL**IAYLKQATK**	2/4
H2-IE^k^	1FNG	1KTD	RDL**IAYLKQASA**K	2/5
H2-IE^k^	1FNG	1R5V	ADL**IAYPKAATK**F	1/5
H2-IE^k^	1FNG	1R5W	ADL**IAYFKAATK**F	2/5

Asterisks indicate self-docking results. The core segment is underlined in the peptide sequence.

An analysis of the peptide-MHC complexes with incorrectly predicted binding registers showed that generally the predicted binding affinity for the correct register was close to that in the predicted predominant register (*i.e.* with highest binding affinity). Furthermore, although experimental binding affinities are not available for each individual binding register, they can be estimated using the RTA sequence-based method [26] for the allotypes with sufficient available binding data. Two incorrectly predicted human complexes are covered by the RTA method, the 1AQD peptide binding to DRB1*0101 (1KLU) and the 1FV1 peptide binding to DRB5*0101 (1FV1). Interestingly, RTA predicts that the binding affinity of the 1AQD peptide in the incorrectly predicted register (FLRGYHQYA), 10.4 kcal/mol, is only slightly lower than that in the correct register observed in the X-ray structure (WRFLRGYHQ), 10.9 kcal/mol. This is a possible explanation of why the structure-based prediction method has difficulty determining which of these two registers is the most stable, particularly since the scoring method was trained on RTA predictions for this allotype. It also suggests that the 1AQD peptide may in fact have two alternative binding registers for DRB1*0101, with the observed register favored by the crystallization environment. Such multiple binding registers have been computationally predicted to be fairly prevalent in strong binders [26], [34] and have also been experimentally observed [48], [49], [50]. The RTA results for the 1FV1 peptide binding to DRB5*0101 shows a similar trend, with the binding affinity of the incorrectly predicted register (VHFFKNIVT), 8.76 kcal/mol, the second highest just after the correct register (FKNIVTPRT), 10.5 kcal/mol, although the affinity difference is greater than for 1AQD. Interestingly, the prediction accuracy for the peptide binding register did not seem to be significantly correlated with the docking accuracy. This is consistent with the above observation that the largest docking errors can be attributed to residues with large side chains that have few contacts with the MHC protein and so are unlikely to have a significant effect on the binding affinity prediction accuracy.

### Comparison to sequence-based approaches

The recent study by Zhang *et al.* [35] evaluated three different structure-based epitope prediction methods on a set of peptides binding to DRB1*0101 and arrived at a rather pessimistic view of such *ab initio* methods, which do not make use of any experimental peptide-MHC binding data. This conclusion was based on the observation that all three methods achieved results that were significantly better than random but still were substantially lower than the best performing sequence-based methods. Our prediction method would not be considered as *ab initio* by the definition used in that study, since the scoring method is parameterized using experimental binding data, albeit for an MHC allotype with completely different peptide binding preferences. We were able to perform a similar comparison to prediction results from our MultiRTA sequence-based method [30] for the two MHC allotypes covered by the HLA-DR and HLA-DP models. In order to avoid overfitting, binding data for the MHC allotype of interest was omitted from the training data used to fit the MultiRTA prediction model. The results for the DRB1*0101 and DPA1*0103/DPB1*0201 data sets are presented in **Table 6**. As expected, the AUC values for the MultiRTA sequence-based method, shown in **Table 6**, are higher than the corresponding values for the structure-based method, shown in **Table 3**, indicating its higher accuracy for these three allotypes. We next discuss the implications of these results for the structure-based and sequence-based approaches to epitope prediction.

**Table 6:** MultiRTA sequence-based prediction results for the same data sets used to obtain the structure-based prediction results in Table 3.

	AUC		
MHC allotype	Peptide	Estimated Core 1	Estimated Core 1–2
DRB1*0101	0.782	0.931	0.910
DPA1*0103/DPB1*0201	0.929	0.988	0.985

The estimated core AUC values were obtained as described in the **Table 3** caption and the Methods section.

## Discussion

The prediction results for a diverse set of representative class II MHC allotypes demonstrate that our method can discriminate binders from non-binders and supports the generality of the structure-based approach to epitope prediction. As in the recent study by Zhang et al. [35], we also found that the prediction performance of the structure-based approach is generally lower than that of sequence-based approaches. This should be expected considering that sequence-based methods generally rely on far more input parameters than structure-based methods in order make binding predictions and also are directly fit to experimental binding data for a similar or identical MHC allotype. For example, the structure-based method presented here uses 27 input parameters for scoring whereas a PSSM sequence-based approach potentially has up to 180 parameters and multi-allotype models, such as NetMHCIIpan and MultiRTA, have even more parameters. Furthermore, the sequence-based results were obtained using prediction models that were trained on binding data for MHC types whose peptide binding preferences are similar enough that even a prediction model trained only on data for the single closest allotype attains relatively high accuracy [29], [30]. Most importantly, although the structure-based approach cannot attain the level of accuracy of the best sequence-based methods, they are able to predict epitopes for MHC allotypes remotely related to allotypes with experimental binding data. Even in principle, this is impossible for a purely sequence-based method. Furthermore, the sequence-based methods that make limited use of structural information, such as peptide-contacting polymorphic MHC residues for NetMHCIIpan [29] and MultiRTA [30] or MHC pockets for TEPITOPE [28], can only interpolate to MHC allotypes that largely share the same MHC polymorphic residues or pockets as experimentally characterized MHC allotypes but in different combinations. For example, the 14 HLA-DR allotypes with sufficient experimental binding data allow fitting a sequence-based model that covers most of the HLA-DR allotypes [29], [30], however these data cannot be used to make predictions for any HLA-DQ allotype, for which little experimental data is available. In summary, each class of prediction method has its optimal domain of applicability. Sequence-based methods should be used for MHC allotypes similar to those that have been experimentally characterized, such as most HLA-DR allotypes. On the other hand, only structure-based methods can currently be used for the large number of remaining allotypes, including almost all of those for HLA-DP, HLA-DQ, and non-human allotypes.

We also derived a rough quantitative estimate of the epitope prediction accuracy for individual 9-mer core segments. Unlike for class I MHC, in which a peptide has only a single binding mode, a peptide can generally bind to class II MHC in multiple binding registers, each defined by which 9-mer segment of the complete peptide contacts the central portion of the binding cleft in the MHC protein. This additional degree of freedom, which cannot be directly ascertained from experimental peptide binding data, makes epitope prediction for class II MHC more difficult than for class I MHC. This is reflected in the generally lower performance of class II MHC prediction methods compared with those for class I MHC, regardless of whether they are sequence-based or structure-based. Using a similar structure-based approach for class I MHC epitope prediction, we previously obtained an AUC of 0.85 for epitope prediction for a H2-K^b^ murine MHC allotype that is highly dissimilar to the human HLA-A*0201 allotype used for training [36]. This AUC value is comparable to the estimated core AUCs in the last column of **Table 3**. In other words, the accuracy for predicting whether or not a peptide binds in a single particular conformation is roughly similar for both class I and class II MHC so that the lower accuracy for class II MHC is predominantly due to an accumulation of errors resulting from the multiple possible peptide binding registers. This suggests a promising application of the structure-based method, discovering strongly binding 9-mer core fragments for uncharacterized MHC allotypes. Presumably the prediction performance would be higher for these fragments because they can only bind in one register. The predicted 9-mer epitopes could then be extended by additional N- and C-terminal residues and experimentally validated.

There are many possible directions for future work. One of the most important is to investigate epitope prediction using homology models of class II MHC allotypes without available X-ray structures in the PDB. Currently, X-ray structures are only available for the human and murine allotypes listed in **Tables 1** and **2**. Different class II allotypes share close sequence identities and relatively conserved backbone structures so that this should be straightforward. The main challenge will be to accurately predict the conformations of the MHC residues that contact the peptide, particularly if a rigid model of the MHC is employed for docking. Several previous studies have investigated class II MHC homology models and their use in docking [32], [51], [52]. Also it is important to attempt to speed up the epitope prediction method. Because we did not attempt to minimize the docking simulation length, it may be possible to significantly reduce it without sacrificing accuracy. Another possible approach is to fit a PSSM to structure-base prediction results for a series of all possible single residue mutants of a strong binding peptide. One other possible improvement is to incorporate MHC flexibility by sampling peptide-contacting MHC residue rotamer conformations or utilizing multiple alternative rigid models of the binding cleft in the docking procedure. Additionally, explicitly accounting for water molecules that have been observed in the peptide-MHC interface [53], [54], [55] may further increase modeling accuracy. Furthermore, as mentioned above, incorporating additional flanking peptide residues in the docking and scoring may improve prediction accuracy. This is supported by observed atomic contacts between flanking residues and the MHC in peptide-MHC complex X-ray structures and improvements in sequence-based epitope prediction methods upon incorporating information on these residues [27], [56]. Adding flanking residues in the docking procedure is straightforward but will increase the simulation convergence time. Also the higher variability in the peptide backbone outside of the central 9-mer core observed in X-ray structures of peptide-MHC complexes implies that these residues will be more difficult to model correctly. It is also worthwhile exploring methods to directly fit the scoring method to experimental peptide-MHC binding affinities rather than relying on error-prone sequence-based prediction results. Finally, one potentially useful application that is well suited to a structure-based approach is to predict peptide epitopes with posttranslational modifications. Experimental evidence indicates that such peptides bind to class II MHC and may have a role in autoimmunity [57], [58].

## References

[1] Todd JA, Bell JI, McDevitt HO (1987). HLA-DQ beta gene contributes to susceptibility and resistance to insulin-dependent diabetes mellitus.. Nature.

[2] Todd JA, Wicker LS (2001). Genetic protection from the inflammatory disease type 1 diabetes in humans and animal models.. Immunity.

[3] Baisch JM, Weeks T, Giles R, Hoover M, Stastny P (1990). Analysis of HLA-DQ genotypes and susceptibility in insulin-dependent diabetes mellitus.. N Engl J Med.

[4] Wordsworth BP, Lanchbury JS, Sakkas LI, Welsh KI, Panayi GS (1989). HLA-DR4 subtype frequencies in rheumatoid arthritis indicate that DRB1 is the major susceptibility locus within the HLA class II region.. Proc Natl Acad Sci U S A.

[5] Fogdell A, Hillert J, Sachs C, Olerup O (1995). The multiple sclerosis- and narcolepsy-associated HLA class II haplotype includes the DRB5*0101 allele.. Tissue Antigens.

[6] Oksenberg JR, Barcellos LF, Cree BA, Baranzini SE, Bugawan TL (2004). Mapping multiple sclerosis susceptibility to the HLA-DR locus in African Americans.. Am J Hum Genet.

[7] Sollid LM, Markussen G, Ek J, Gjerde H, Vartdal F (1989). Evidence for a primary association of celiac disease to a particular HLA-DQ alpha/beta heterodimer.. J Exp Med.

[8] Matsuki K, Grumet FC, Lin X, Gelb M, Guilleminault C (1992). DQ (rather than DR) gene marks susceptibility to narcolepsy.. Lancet.

[9] Mignot E, Lin L, Rogers W, Honda Y, Qiu X (2001). Complex HLA-DR and -DQ interactions confer risk of narcolepsy-cataplexy in three ethnic groups.. Am J Hum Genet.

[10] Seamons A, Sutton J, Bai D, Baird E, Bonn N (2003). Competition between two MHC binding registers in a single peptide processed from myelin basic protein influences tolerance and susceptibility to autoimmunity.. J Exp Med.

[11] Maverakis E, Beech J, Stevens DB, Ametani A, Brossay L (2003). Autoreactive T cells can be protected from tolerance induction through competition by flanking determinants for access to class II MHC.. Proc Natl Acad Sci U S A.

[12] Muller U, Akdis CA, Fricker M, Akdis M, Blesken T (1998). Successful immunotherapy with T-cell epitope peptides of bee venom phospholipase A2 induces specific T-cell anergy in patients allergic to bee venom.. J Allergy Clin Immunol.

[13] Marcotte GV, Braun CM, Norman PS, Nicodemus CF, Kagey-Sobotka A (1998). Effects of peptide therapy on ex vivo T-cell responses.. J Allergy Clin Immunol.

[14] von Garnier C, Astori M, Kettner A, Dufour N, Heusser C (2000). Allergen-derived long peptide immunotherapy down-regulates specific IgE response and protects from anaphylaxis.. Eur J Immunol.

[15] Haselden BM, Kay AB, Larche M (1999). Immunoglobulin E-independent major histocompatibility complex-restricted T cell peptide epitope-induced late asthmatic reactions.. J Exp Med.

[16] Oldfield WL, Larche M, Kay AB (2002). Effect of T-cell peptides derived from Fel d 1 on allergic reactions and cytokine production in patients sensitive to cats: a randomised controlled trial.. Lancet.

[17] Larche M (2006). Immunoregulation by targeting T cells in the treatment of allergy and asthma.. Curr Opin Immunol.

[18] Guan P, Doytchinova IA, Zygouri C, Flower DR (2003). MHCPred: A server for quantitative prediction of peptide-MHC binding.. Nucleic Acids Res.

[19] Hattotuwagama CK, Toseland CP, Guan P, Taylor DJ, Hemsley SL (2006). Toward prediction of class II mouse major histocompatibility complex peptide binding affinity: in silico bioinformatic evaluation using partial least squares, a robust multivariate statistical technique.. J Chem Inf Model.

[20] Nielsen M, Lundegaard C, Worning P, Hvid CS, Lamberth K (2004). Improved prediction of MHC class I and class II epitopes using a novel Gibbs sampling approach.. Bioinformatics.

[21] Murugan N, Dai Y (2005). Prediction of MHC class II binding peptides based on an iterative learning model.. Immunome Res.

[22] Cui J, Han LY, Lin HH, Tang ZQ, Jiang L (2006). MHC-BPS: MHC-binder prediction server for identifying peptides of flexible lengths from sequence-derived physicochemical properties.. Immunogenetics.

[23] Lata S, Bhasin M, Raghava GP (2007). Application of machine learning techniques in predicting MHC binders.. Methods Mol Biol.

[24] Liu W, Wan J, Meng X, Flower DR, Li T (2007). In silico prediction of peptide-MHC binding affinity using SVRMHC.. Methods Mol Biol.

[25] Salomon J, Flower DR (2006). Predicting Class II MHC-Peptide binding: a kernel based approach using similarity scores.. BMC Bioinformatics.

[26] Bordner AJ, Mittelmann HD (2010). Prediction of the binding affinities of peptides to class II MHC using a regularized thermodynamic model.. BMC Bioinformatics.

[27] Nielsen M, Lundegaard C, Lund O (2007). Prediction of MHC class II binding affinity using SMM-align, a novel stabilization matrix alignment method.. BMC Bioinformatics.

[28] Sturniolo T, Bono E, Ding J, Raddrizzani L, Tuereci O (1999). Generation of tissue-specific and promiscuous HLA ligand databases using DNA microarrays and virtual HLA class II matrices.. Nat Biotechnol.

[29] Nielsen M, Lundegaard C, Blicher T, Peters B, Sette A (2008). Quantitative predictions of peptide binding to any HLA-DR molecule of known sequence: NetMHCIIpan.. PLoS Comput Biol.

[30] Bordner AJ, Mittelmann HD (2010). MultiRTA: A simple yet reliable method for predicting peptide binding affinities for multiple class II MHC allotypes.. BMC Bioinformatics.

[31] Peters B, Sidney J, Bourne P, Bui HH, Buus S (2005). The immune epitope database and analysis resource: from vision to blueprint.. PLoS Biol.

[32] Davies MN, Sansom CE, Beazley C, Moss DS (2003). A novel predictive technique for the MHC class II peptide-binding interaction.. Mol Med.

[33] Schafroth HD, Floudas CA (2004). Predicting peptide binding to MHC pockets via molecular modeling, implicit solvation, and global optimization.. Proteins.

[34] Tong JC, Zhang GL, Tan TW, August JT, Brusic V (2006). Prediction of HLA-DQ3.2beta ligands: evidence of multiple registers in class II binding peptides.. Bioinformatics.

[35] Zhang H, Wang P, Papangelopoulos N, Xu Y, Sette A (2010). Limitations of Ab initio predictions of peptide binding to MHC class II molecules.. PLoS One.

[36] Bordner AJ, Abagyan R (2006). Ab initio prediction of peptide-MHC binding geometry for diverse class I MHC allotypes.. Proteins.

[37] Abagyan R, Totrov M (1994). Biased probability Monte Carlo conformational searches and electrostatic calculations for peptides and proteins.. J Mol Biol.

[38] Momany FA, McGuire RF, Burgess AW, Scherage HA (1975). Energy parameters in polypeptides. VII. Geometric parameters, partial atomic charges, nonbonded interactions, ydrogen bond interactions, and intrinsic torsional potentials for the naturally occurring amino acids.. J Phys Chem.

[39] Nemethy GN, Pottle MS, Scheraga HA (1983). Energy parameters in polypeptides. 9. Updating of gemetrical parameters, nonbonded interactions and hydrogen bond interactions for the naturally occurring amino acids.. J Phys Chem.

[40] Nemethy GN, Gibson KD, Palmer KA, Yoon CN, Paterlini G (1992). Energy parameters in polypeptides. 10. Improved geometrical parameters and nonbonded interactions for use in the ECEPP/3 algorithm, with application to proline-containing peptides.. J Phys Chem.

[41] Fernandez-Recio J, Totrov M, Abagyan R (2002). Soft protein-protein docking in internal coordinates.. Protein Sci.

[42] Breiman L (2001). Random forests.. Machine Learning.

[43] Betancourt MR, Thirumalai D (1999). Pair potentials for protein folding: choice of reference states and sensitivity of predicted native states to variations in the interaction schemes.. Protein Sci.

[44] Zhang C, Liu S, Zhou H, Zhou Y (2004). An accurate, residue-level, pair potential of mean force for folding and binding based on the distance-scaled, ideal-gas reference state.. Protein Sci.

[45] Wang G, Dunbrack RL (2003). PISCES: a protein sequence culling server.. Bioinformatics.

[46] Sidney J, Steen A, Moore C, Ngo S, Chung J (2010). Five HLA-DP molecules frequently expressed in the worldwide human population share a common HLA supertypic binding specificity.. J Immunol.

[47] Li W, Godzik A (2006). Cd-hit: a fast program for clustering and comparing large sets of protein or nucleotide sequences.. Bioinformatics.

[48] Fairchild PJ, Pope H, Wraith DC (1996). The nature of cryptic epitopes within the self-antigen myelin basic protein.. Int Immunol.

[49] McFarland BJ, Sant AJ, Lybrand TP, Beeson C (1999). Ovalbumin(323–339) peptide binds to the major histocompatibility complex class II I-A(d) protein using two functionally distinct registers.. Biochemistry.

[50] Anderton SM, Viner NJ, Matharu P, Lowrey PA, Wraith DC (2002). Influence of a dominant cryptic epitope on autoimmune T cell tolerance.. Nat Immunol.

[51] Tong JC, Bramson J, Kanduc D, Chow S, Sinha AA (2006). Modeling the bound conformation of Pemphigus vulgaris-associated peptides to MHC Class II DR and DQ alleles.. Immunome Res.

[52] Simon A, Simon I, Rajnavolgyi E (2002). Modeling MHC class II molecules and their bound peptides as expressed at the cell surface.. Mol Immunol.

[53] Stern LJ, Brown JH, Jardetzky TS, Gorga JC, Urban RG (1994). Crystal structure of the human class II MHC protein HLA-DR1 complexed with an influenza virus peptide.. Nature.

[54] Murthy VL, Stern LJ (1997). The class II MHC protein HLA-DR1 in complex with an endogenous peptide: implications for the structural basis of the specificity of peptide binding.. Structure.

[55] Wilson N, Fremont D, Marrack P, Kappler J (2001). Mutations changing the kinetics of class II MHC peptide exchange.. Immunity.

[56] Nielsen M, Lund O (2009). NN-align. An artificial neural network-based alignment algorithm for MHC class II peptide binding prediction.. BMC Bioinformatics.

[57] Engelhard VH, Altrich-Vanlith M, Ostankovitch M, Zarling AL (2006). Post-translational modifications of naturally processed MHC-binding epitopes.. Curr Opin Immunol.

[58] Doyle HA, Mamula MJ (2001). Post-translational protein modifications in antigen recognition and autoimmunity.. Trends Immunol.

